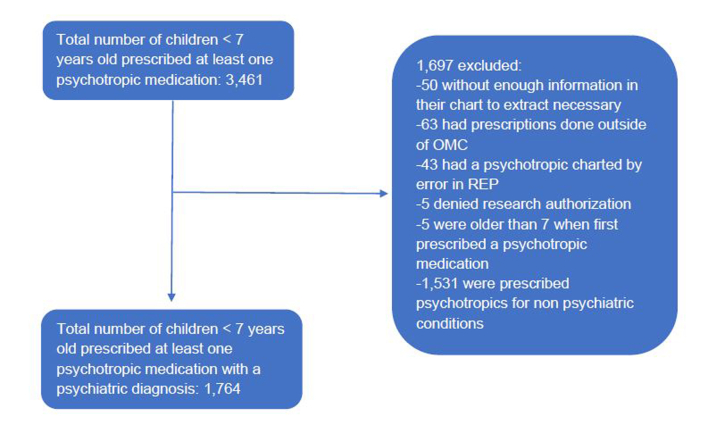# Impact of Social Determinants of Health and Professional Guidelines for Pharmacotherapy and Psychotherapy Recommendations for the Treatment of Young Children: A Retrospective Study

**DOI:** 10.1016/j.jaacop.2024.09.010

**Published:** 2024-12-11

**Authors:** Chris Wang, Maria Saliba, Kierstin S. Utter, Joshua Wy, Alex S. Roth, Juan F. Garzon Hincapie, Tatsumi Yanaba, Pedro Versuti Del Cioppo Vasques, Vanessa K. Pazdernik, Chung-Il Wi, Monica J. Taylor-Desir, Paul E. Croarkin, Magdalena Romanowicz

**Affiliations:** a NYU Grossman School of Medicine, New York, New York; b Mayo Clinic, Rochester, Minnesota; c Kaiser Permanente, Pleasanton, California

**Keywords:** early childhood, health care disparities, psychopharmacology, psychotherapy

## Abstract

**Objective:**

To examine pharmacotherapy and psychotherapy treatment recommendations among different races and socioeconomic groups of young children. A secondary objective evaluated whether changes in the 2007 American Academy of Child and Adolescent Psychiatry (AACAP) guidelines for attention-deficit/hyperactivity disorder (ADHD) treatment affected community prescribing practices. Hypotheses were that non-White children would be less likely to have psychotherapeutic treatments recommended for mental health issues and children with lower socioeconomic status index scores would be less likely to receive a psychotropic medication prescription.

**Method:**

The sample included 1,764 children ages 7 years or younger prescribed at least 1 psychotropic medication for psychiatric indications between 2003 and 2019 identified through the Rochester Epidemiology Project. Data were examined with χ^2^ test and logistic regression models.

**Results:**

The sample included 29% female, 1.6% Asian, 6.9% Black, 5.3% Hispanic, 7.7% other race/ethnicity, and 79% White participants. The most common psychiatric diagnosis was attention-deficit/hyperactivity disorder (91.6%). The majority of first prescriptions of psychotropic medications were written by primary care physicians or pediatricians (69%) rather than psychiatrists. Recommendations were not affected by patient socioeconomic status, race/ethnicity, or insurance (all *p* > .18). Following 2007 AACAP guideline updates, there was a significant increase in psychotherapy recommendations for children diagnosed with attention-deficit/hyperactivity disorder. For children younger than age 6, the rate rose from 54.4% to 65.5% after 2007 (*p* = .002), and for children 6 to 7, it increased from 45.7% to 52.7% (*p* = .03).

**Conclusion:**

Children’s socioeconomic status, race/ethnicity, and insurance did not affect treatment recommendations of clinicians. However, children whose first prescription of psychotropic medication was from a primary care physician or pediatrician were less likely to have a recommendation for psychotherapy compared with children who were seen by psychiatrists.

**Diversity & Inclusion Statement:**

One or more of the authors of this paper self-identifies as a member of one or more historically underrepresented racial and/or ethnic groups in science. One or more of the authors of this paper self-identifies as a member of one or more historically underrepresented sexual and/or gender groups in science. We actively worked to promote sex and gender balance in our author group.

## Plain language summary

This study examined how treatment recommendations for attention-deficit/hyperactivity disorder (ADHD) varied across different racial and socioeconomic groups of young children. Data from 1,764 children aged 7 and under, who were prescribed at least one psychotropic medication between 2003 and 2019, were analyzed. The authors found that treatment recommendations were not influenced by race, socioeconomic status, or insurance, but that psychotherapy recommendations for ADHD in preschoolers were somewhat increased after updates to the 2007 AACAP guidelines for ADHD were published. Children seen by their family physician or pediatrician who received their first ADHD medication were less likely to receive psychotherapy recommendations compared to those seen by psychiatrists.

Disruptive behavior disorders are one of the most common reasons for psychiatric referrals and prescriptions of stimulant medications, mood stabilizers, and antipsychotics.^1^, ^2^, ^3^, ^4^, ^5^ Prior work suggests that pharmacologic treatment is often prescribed for disruptive behavior disorders in young children without accompanying psychosocial interventions,^6^ despite substantial research supporting behavioral parent training as the leading treatment for disruptive behaviors^7^, ^8^, ^9^ and recommendations issued in 2007 by the American Academy of Child and Adolescent Psychiatry (AACAP) for the use of psychotherapeutic interventions before pharmacologic treatment.^10^

Despite the increase in psychiatric diagnoses of young children, there is limited research on access to evidence-based treatments as well as the quality of mental health care provided for Black and Hispanic children ages 7 and younger.^11^ Without early and effective interventions, common emotional-behavioral problems in young children often lead to lifelong impairment.^6^ Studies suggest that Black and Hispanic children have a higher prevalence of mental and behavioral health conditions but are less likely to receive high-quality mental health care compared with their White peers.^12^ Additionally, Black and Hispanic youth have a lower number of mental health care visits and are less likely to be referred to a mental health care specialist compared with their White peers.^13^^,^^14^ In adolescents, there also seems to be a discrepancy in the types of psychotropic medications prescribed for different racial groups. A study done by Cook *et al.*^15^ showed that White children are 2 to 9 times more likely to receive stimulant medications, 4 times more likely to receive antidepressants, and 2 times more likely to receive antipsychotic medications compared with Black and Latino teens. These disparities in mental health care use and pharmacotherapy persisted over time for both Black and Latino youth compared with their White counterparts.^16^ Efforts focused on understanding and increasing access to mental health clinicians and evidence-based pharmacotherapy for resource-limited youth are critically needed.

The goal of this study was to expand the evidence base of behavioral and pharmacologic treatment recommendations and their implementation in young children through 3 main aims and objectives. First, we retrospectively examined trends in behavioral and pharmacologic treatment recommendations and access to care in children ages 0 to 7 years 11 months through the study of a birth cohort by evaluating the differences in prescribing practices for patients of different sex, race, ethnicity, socioeconomic status (SES), and insurance type. We hypothesized that children of Black and Hispanic race/ethnicity, with lower SES, and with public insurance would be less likely to receive treatment for mental health issues evidenced by lower rates of psychotropic medications and psychotherapy recommended to them compared with their White peers.

Second, in 2007, the AACAP issued guidelines for the treatment of attention-deficit/hyperactivity disorder (ADHD) in young children (ages 0-6 years). The AACAP recommended at least an 8-week trial of evidence-based behavioral therapy, such as parent management training (PMT), before consideration of a medication trial.^10^^,^^17^ For our second aim, we sought to examine whether the implementation of treatment guidelines for young children as described by AACAP was reflected in the community prescribing practices over time. We hypothesized that there would be an increase in psychotherapy treatment recommendations in children with ADHD and other disruptive, externalizing behaviors, during the 2008-2019 study period (time after the AACAP guidelines update) by comparing data from 2003 to 2007 and from 2008 to 2019.

Third, we sought to see if there are any differences in prescribing patterns and psychotherapy recommendations between psychiatrists and primary care physicians (PCPs). We hypothesized that psychiatrists would have increased rates of pharmacotherapy prescriptions and psychotherapy recommendations than their PCP counterparts.

## Method

### Study Design

This retrospective cohort study examined data from the Rochester Epidemiology Project (REP) of children prescribed at least 1 psychotropic medication during their lifetime between January 1, 2003, and December 31, 2019. The REP medical records linkage system was established in 1966 to capture health care information for the entire population of Olmsted County, Minnesota, from both urban and rural areas in the Midwest region of the United States. The data available electronically include demographic characteristics, medical diagnostic codes, procedure and service, and medication groups.^18^ In addition, for each resident, the system keeps a complete list of all paper records, electronic records, and scanned documents that are available in full text for in-depth review and abstraction.^19^ We followed the Strengthening the Reporting of Observational Studies in Epidemiology (STROBE) reporting principles when writing this manuscript (Table S1, available online).^20^ The study was approved by both the Mayo Clinic and the Olmsted Medical Center institutional review boards before any research activities were undertaken.

### Participants

All children whose parents had consented to research with an authorization on record were eligible, and children whose parents had not consented were excluded. The research team identified 2,526 unique patients ages 0 to 7 years who were prescribed at least 1 psychotropic medication between January 1, 2003, and December 31, 2019. Children (n = 762) with psychotropic medication prescriptions to target nonpsychiatric problems (eg, seizures, headaches, pain management, apnea of prematurity) were excluded. None of the patients were excluded due to cardiac issues. A total of 1,764 children having at least 1 psychotropic medication prescription for a primary psychiatric reason were selected for the study (Figure S1, available online). Of included patients, 35 had comorbid cardiac conditions, and it was ensured that α-agonists and/or other examined medications were not prescribed specifically for cardiac issues.

### Measures

Demographic data available from the electronic medical record included biological sex, age, race, ethnicity, insurance, home zip codes, and county of residence. As maternal education, income, and insurance status data were not available in chart review for many patients, the SES was calculated with the Housing-Based Socioeconomic Status (HOUSES) index, a validated individual-level SES measuring program developed for Olmsted County. The HOUSES index geocodes addresses (zip codes with accompanying counties) available from study participants and matches them to real property data of individual housing units from the county assessor’s office.^21^^,^^22^ The program uses 4 property variables, including housing value, square footage of the housing unit, number of bedrooms, and number of bathrooms. All data are formulated into a standardized HOUSES score through summation (*z* score) for each variable. The higher the HOUSES index (*z* score), the higher the SES. We obtained the patients’ addresses at index date (first psychotropic prescription date) and matched them to the HOUSES index. There were 1,284 children who qualified for the analysis.

For treatment-related comparisons, we coded for the number and types of psychotropic medications, including stimulants; nonstimulants (atomoxetine, guanfacine, and clonidine); antidepressants (eg, selective serotonin reuptake inhibitors, serotonin and norepinephrine reuptake inhibitors); antipsychotics; mood stabilizers; or benzodiazepines, anxiolytics, and hypnotics. Psychiatric diagnoses were defined by disorders listed in *DSM-5*. We also gathered data including whether the first prescription of psychotropics to these children was by a psychiatrist or aPCP and each child’s psychiatric history (eg, number of psychiatric hospitalizations), history of self-injury (eg, head banging, cutting), or suicidal behavior (eg, overdose).

We also closely examined psychotherapy recommendations, including whether psychotherapy treatment was recommended and the date. Data were also coded for psychotherapy treatment recommended before or after starting psychotropic medications and whether the patient only started therapy without completion or completed the therapy. The cause of not starting therapy for applicable cases was also noted (eg, high cost, anticipated long duration of therapy, poor result from past experiences, patient/family preference without a stated reason). For each therapy recommended, we coded the modalities: behavioral management, PMT, parent–child interaction therapy, play therapy, parenting class, applied behavior analysis, social skills, occupational therapy, speech therapy, cognitive-behavioral psychotherapy, and music therapy. To allow comparisons with other studies, we also considered a stricter definition of psychotherapy modalities that included only the behavioral management, PMT, parent–child interaction therapy, and cognitive-behavioral psychotherapy modalities. Secondarily, we compared data from 2003 to 2007 and from 2008 to 2019 in 2 groups to see whether the 2007 AACAP guidelines impacted community prescribing practices.

We coded for each psychiatric diagnosis that was consistently carried forward from the first intake interview. For example, the differential diagnosis during the initial intake diagnostic assessment can often be broad. As such, we took the consistent, solidified psychiatric diagnosis from reviewing notes from the next several follow-up visits. Furthermore, we also coded for demographic data available from the electronic medical record including biological sex, age, race, past psychiatric history, past medical history, and trauma (eg, family conflicts such as divorce or separation from parents, physical abuse, medical trauma, sexual trauma). Finally, for family history of psychiatric disorders, we coded for first-degree relatives (ie, mother, father, full siblings).

### Statistical Analysis

Continuous and categorical data were reported as median with interquartile range and minimum and maximum and counts with percentages, respectively. The χ^2^ test was used to assess associations between insurance, race/ethnicity groups, and SES. Logistic regression analyses were used to assess differences in trauma and trauma subtypes between race/ethnicity groups and SES. Logistic regression analyses were also used to assess differences in rates of psychotherapy treatment recommendations between insurance, race/ethnicity groups, SES, sex, childhood trauma, family history, and prescribing physician’s medical specialty. We also assessed differences in starting psychotherapy between medical specialty of the prescribing physician. We compared data for patients whose first psychotropic medication was prescribed on or before December 31, 2007, with data from after this date to evaluate the effect of AACAP guidelines on prescribing practices. This included evaluating psychotherapy recommendation rates, the timing of these recommendations relative to the psychotropic medication prescription, and the usage rates of various psychotropic medications among children diagnosed with ADHD, ages 2 to 5 and 6 to 7. For all analyses with psychotherapy treatment recommendations, we considered an inclusive definition across all therapy modalities as well as both a stricter definition that included only the behavioral management, PMT, parent–child interaction therapy, and cognitive-behavioral psychotherapy modalities and one that included the other modalities (play therapy, parenting class, applied behavior analysis, social skills, occupational therapy, speech therapy, and music therapy). All analyses were conducted after excluding missing data. To assess its impact, we compared rates of psychotherapy treatment between groups with available vs missing demographic information. R version 4.2.2 (R Foundation for Statistical Computing, Vienna, Austria) was used for statistical analysis. A *p* value < .05 was considered significant.

## Results

### Background Characteristics

The full sample size was 1,764 children, with an age range of 2 to 7 years and a median age of 6 years. The majority of the children were 6 to 7 years old (69%) when they were prescribed a psychotropic medication for the first time (Table 1). Regarding HOUSES index (calculated on 1,284 children with sufficient data), the sample was almost equally distributed around the 4 SES quartiles, with 455 (30.3%) of the sample belonging to the 4th quartile, corresponding to the highest SES. Most children (958 [54.3%]) met criteria for 1 current psychiatric diagnosis, 12 (0.7%) had zero diagnoses, and the rest had 2 or more diagnoses (794 [45.0%]), with ADHD being the overall most prevalent disorder (1,615 [91.6%]) (Table 1). In the cohort, 66.9% had a first-degree relative with a history of mental illness, with depressive disorders (35.7%), ADHD (33.9%), and anxiety disorders (26.4%) being the most common diagnoses.

**Table 1:** Demographic Data for Study Patients

Demographic data	Full sample (N = 1,764)		
	n	(%)	
Insurance^a^			
Public	455	(30.3)	
Private	970	(64.5)	
None	79	(5.3)	
	**Median**	**(IQR)**	**[range]**
Age when first prescribed a psychotropic, y	6	(5, 7)	[2, 7]
	**n**	**(%)**	
2-5	550	(31.2)	
6-7	1,214	(68.8)	
First medication prescribed			
2007 or earlier	567	(32.1)	
After 2007	1,197	(67.9)	
Race/ethnicity^b^			
Asian	28	(1.6)	
Black	122	(6.9)	
Hispanic	93	(5.3)	
Multiracial	12	(0.7)	
Native American	10	(0.6)	
Native Hawaiian or Pacific Islander	2	(0.1)	
Other/unknown	112	(6.3)	
White	1,385	(78.5)	
Sex assigned at birth			
Female	509	(28.9)	
Male	1,255	(71.1)	
First prescription of psychotropics by provider^c^			
PCP/pediatrician	1,204	(68.9)	
Psychiatrist	543	(31.1)	
HOUSES index quartile^d^			
Q1	284	(22.1)	
Q2	289	(22.5)	
Q3	321	(25.0)	
Q4	390	(30.4)	
Diagnosis			
ADHD	1,615	(91.6)	
Emotional behavioral disturbance	325	(18.4)	
Neurodevelopmental disorder	262	(14.9)	
Sleep disturbance, parasomnia, hypersomnia	201	(11.4)	
Anxiety disorders	173	(9.8)	
Elimination disorder^e^	100	(5.7)	
Parent–child relational issue	83	(4.7)	
Adjustment disorder	67	(3.8)	
Autism spectrum disorder	56	(3.2)	
Sibling rivalry	32	(1.8)	
Trauma- and stress-related disorders^f^	29	(1.7)	
Depressive disorders	26	(1.5)	
Disruptive mood dysregulation disorder	9	(0.5)	
Bipolar disorder	3	(0.2)	
Eating disorder	1	(0.1)	
Gender identity disorder^e^	0	(0.0)	
	**Median**	**(IQR)**	**[range]**
No. of comorbid diagnoses	1	(1, 2)	[0, 7]
	**n**	**(%)**	
0	12	(0.7)	
1	958	(54.3)	
2 or more	794	(45.0)	

Note: ADHD = attention-deficit/hyperactivity disorder; HOUSES = Housing-based Socioeconomic Status; IQR = interquartile range; PCP = primary care physician; Q1-Q4 = quartile 1–quartile 4.

a n = 1,504 because 260 were missing.

b Hispanic category includes all who indicated Hispanic or Latino ethnicity or any Spanish origin (Mexican, Mexican American, Chicano, Puerto Rican, Cuban, or other Hispanic or Latino origin).

c n = 1,747 because 2 were missing and 15 were other specialty.

d n = 1,284 because 480 were missing.

e n = 1,763 because 1 was missing.

f n = 1,705 because 59 were missing.

### Race and SES

**Figure 1:**
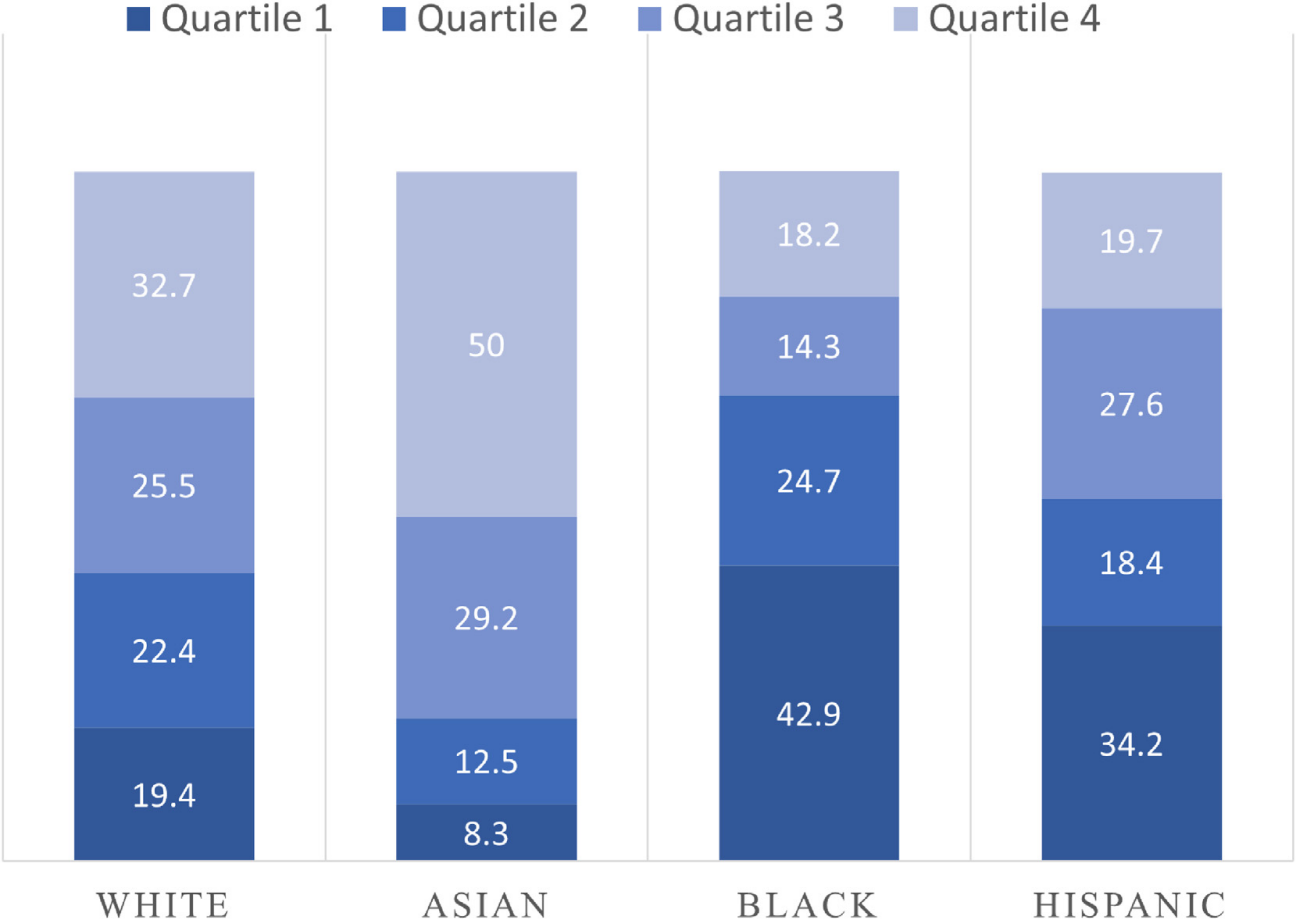
Distribution of Socioeconomic Status Quartiles Among Different Racial and Ethnic Groups Based on the Housing-Based Socioeconomic Status (HOUSES) Index

The analysis of insurance coverage among different racial and ethnic groups showed statistically significant differences (*p* < .001). Non-Hispanic White and Asian children were mostly covered by private insurance, 829 (72.7%) and 20 (83.3%), respectively, whereas Black and Hispanic children were mostly covered by public insurance, 51 (56%) and 41 (51.9%), respectively. Similar statistically significant disparities were seen in the analysis of SES levels among different racial and ethnic groups (*p* < .001), with most White and Asian children being in the highest SES level (fourth quartile), 32.7% and 50.0%, respectively, and most Black and Hispanic children being in the lowest SES level (first quartile), 42.9% and 34.2%, respectively (Figure 1).

### Trauma: Racial and Ethnic Groups and SES

Trauma was reported in 24.5% (431/1,761) of children, with family conflicts being the most common subtype among children with trauma (56.1% [242/431]). Reported trauma was most common among Black (54 [44.3%]) and Hispanic (32 [34.4%]) children compared with White (297 [21.5%]) and Asian (5 [17.9%]) children. More trauma overall (33.2% [94/284]) and family trauma subtype among those with trauma (71.3% [67/94]) were reported in children belonging to the SES first quartile, which represents the lowest SES level, compared with the higher SES quartiles (both *p* ≤ .01).

### Psychotherapy Recommendations: Racial and Ethnic Groups, SES, Sex, Trauma, Family History, and Provider Type

There were no significant differences in psychotherapy treatment recommendations between different racial and ethnic groups (*p* = .52). Of 1,628 children, psychotherapy was recommended for 883 (54.2%). Among White children, the recommendation rate was 54.2% (751/1,385), whereas it was 53.6% (15/28) among Asian, 50.0% (61/122) among Black, and 60.2% (56/93) among Hispanic children. Similarly, differences in psychotherapy treatment recommendations based on SES and insurance coverage were not statistically significant (both *p* ≥ .18) (Table 2). There were also no significant differences in rates of recommendations of strict psychotherapy modalities and other psychotherapy modalities across these sociodemographics (all *p* ≥ .19). However, psychotherapy was more likely to be recommended to boys (702 [56%] vs 252 [50%]; *p* = .01), children with childhood trauma (262 [61%] vs 692 [52%]; *p* = .001), and children with a family history of mental illness (680 [59%] vs 262 [46%]; *p* < .001) compared with their counterparts. These differences were revealed to be driven by recommendations of the strict psychotherapy modalities based on sex and family history of mental illness (both *p* ≤ .02) and unspecified modalities based on childhood trauma (*p* < .001). Further, children whose first prescription of psychotropic medications came from psychiatrists were more likely to have psychotherapy recommended compared with children who saw PCPs (364 [67%] and 583 [48%], respectively; *p* < .001) and were more likely to start treatment (281 [77%] and 402 [69%], respectively; *p* = .005).

**Table 2:** Difference in Psychotherapy Recommendations Between Demographic Characteristics

Characteristic	No.	Psychotherapy recommended		*p*	Strict psychotherapy		*p*	Other therapy modalities		*p*
		n	(%)		n	(%)		n	(%)	
Full sample	1,764	954	(54.1)		658	(37.3)		142	(8.0)	
Race/ethnicity^a^				.52			.83			.95
Asian	28	15	(53.6)		10	(8.0)		2	(7.1)	
Black	122	61	(50.0)		41	(33.6)		10	(8.2)	
Hispanic	93	56	(60.2)		35	(38.7)		9	(9.7)	
White	1,385	751	(54.2)		521	(37.6)		111	(8.0)	
HOUSES index quartile^b^				.18			.90			.47
Q1	284	168	(59.2)		113	(39.8)		20	(7.0)	
Q2	289	152	(52.6)		107	(37.0)		25	(8.7)	
Q3	321	169	(52.6)		121	(37.7)		28	(8.7)	
Q4	390	228	(58.5)		152	(39.0)		41	(10.5)	
Insurance^c^				.32			.19			.37
Private	970	554	(57.1)		393	(40.5)		86	(8.9)	
Public	455	247	(54.3)		168	(36.9)		34	(7.5)	
Sex assigned at birth				.01			.02			.24
Female	509	252	(49.5)		168	(33.0)		35	(6.9)	
Male	1,255	702	(55.9)		490	(39.0)		108	(8.5)	
Trauma^d^				.001			.57			.65
Yes	431	262	(60.8)		166	(38.5)		37	(8.6)	
No	1,330	692	(52.0)		492	(37.0)		105	(7.9)	
Family history of mental illness^e^				<.001			<.001			.38
Yes	1,158	680	(58.7)		479	(41.4)		97	(8.4)	
No	572	262	(45.8)		174	(30.4)		41	(7.2)	
First prescription of psychotropics by provider^f^				<.001			<.001			.01
PCP/pediatrician	1,204	583	(48.4)		400	(33.2)		83	(6.9)	
Psychiatrist	543	364	(67.0)		253	(46.6)		57	(10.5)	

Note: Strict psychotherapy included therapy modalities that were examined by Gleason et al.^10^ (behavioral management, parent management training, parent–child interaction therapy, and cognitive-behavioral psychotherapy). Other therapy modalities included those that were reviewed for their impact on early childhood psychiatric disorders (play therapy, parenting class, applied behavior analysis, social skills, occupational therapy, speech therapy, and music therapy). The number of children that were recommended a strict and non-strict psychotherapy definition do not necessarily add up to 100% for 2 reasons: patients could be recommended both a strict and a non-strict psychotherapy treatment type and thus would be represented in both counts (n = 8), and some patients were recommended psychotherapy treatment, but 1 type was not specified and therefore they were not part of either count (n = 162). HOUSES = Housing-based Socioeconomic status; PCP = primary care physician; Q1-Q4 = quartile 1–quartile 4.

a n = 1,628 because 136 were excluded.

b n = 1,284 because 480 were missing.

c n = 1,425 because 260 were missing and 79 had none.

d n = 1,761 because 3 were missing.

e n = 1,730 because 34 were missing.

f n = 1,747 because 2 were missing and 15 were other specialty.

The differences in recommendations for both strict psychotherapy modalities and other psychotherapy modalities were higher among psychiatrists (both *p* ≤ .01). To account for potential confounding between clinical risk factors (such as trauma and family history of mental illness) and provider influence on psychotherapy recommendations, we performed a multivariable logistic regression for more nuanced interpretation. After adjusting for sex and clinical risk factors, recommendations from psychiatrists remained significantly higher for any psychotherapy, strict psychotherapy, and other therapy modalities (odds ratios 2.03, 1.65, and 1.61; all *p* ≤ .01) (Table S2, available online). There was an independent association of boys and children with family history of mental illness with recommendations for both any psychotherapy and strict psychotherapy (all *p* ≤ 0.01), whereas trauma was linked only to recommendation for unspecified psychotherapy modalities (*p* < .001).

Psychotherapy recommendation rates were notably lower among children with missing HOUSES index values (n = 480; 49.4%) compared with children with complete HOUSES index quartiles. Similarly, children with missing family history of mental illness (n = 34; 35.3%) exhibited lower recommendation rates compared with the children with family history of mental illness.

### AACAP 2007 Guidelines and Timing of Therapy Recommendations

Among children younger than 6 years old diagnosed with ADHD (n = 482), the frequency of psychotherapy recommendations increased from 54.4% in 2007 or earlier to 65.5% after 2007 (*p* = .02). Recommendations for strict psychotherapy appeared to have similar rates (46.2%-45.7%; *p* = .90), whereas the rates were trending higher for other psychotherapy modalities (5.6%-10.2%; *p* = .08) and unspecified modalities (3.8%-10.9%; *p* = .03). We examined the potential interaction between age groups (2-5 vs 6-7) and 2007 or earlier vs after 2007 on psychotherapy recommendations for children diagnosed with ADHD and found that the increase in rates from 2007 to post-2007 was not significantly different between the age groups (*p* = .43). We see a similar pattern among children 6 to 7 years old (n = 1,133), with rates increasing from 45.7% to 52.7% (*p* = .03) overall, but these appear to be also primarily driven by other psychotherapy modalities (3.9%-7.8%; *p* = .009) and unspecified modalities (6.3%-10.7%; *p* = .01). Similar patterns were seen in White children, whereas data to assess trends in Black and Hispanic children were limited (Table 3). Contrarily, after publication of the 2007 AACAP guidelines, though not statistically significant, a higher percentage (49.3%) of children younger than 6 years old received therapy recommendations at the same time or after their psychotropic medication prescription than before (39.1%) their first psychotropic medication prescription (Table 3). Prescription of stimulants decreased slightly in both age groups (from 98.1% to 92.2% in <6 and from 99.4% to 96.9% in 6-7; both *p* ≤ .004), whereas melatonin prescription increased (from 3.8% to 10.6% in <6 and from 0.3% to 4.0% in 6-7; both *p* ≤ .007).

**Table 3:** Difference in Psychotherapy Characteristics Between Time Eras Among Children Diagnosed With Attention-Deficit/Hyperactivity Disorder (n = 1,615)

Age group	Characteristic	2007 or earlier		After 2007		*p*
2 to 5 y (24 to <72 mo)		n = 160		n = 322		
		**n**	**(%)**	**n**	**(%)**	
	Psychotherapy recommendation	87	(54.4)	211	(65.5)	.02
	Black	10	(66.7)	16	(61.5)	.74
	Hispanic	3	(60.0)	12	(57.1)	.91
	White	66	(52.4)	160	(65.0)	.02
	Psychotherapy recommendation relative to psychotropic					.11
	Before psychotropic	53	(60.9)	107	(50.7)	
	Same time or after psychotropic	34	(39.1)	104	(39.1)	
6 to 7 y (72 to <96 mo)		n = 363		n = 770		
		**n**	**(%)**	**n**	**(%)**	
	Psychotherapy recommendation	166	(45.7)	406	(52.7)	
	Black	7	(29.2)	23	(47.9)	.12
	Hispanic	8	(66.7)	28	(59.6)	.65
	White	136	(46.7)	325	(53.5)	.06
	Psychotherapy recommendation relative to psychotropic^a^					.35
	Before psychotropic	90	(54.5)	201	(50.2)	
	Same time or after psychotropic	75	(45.5)	199	(49.8)	

**Note:**

a n = 166 for children ages 6 to 7 who received a psychotherapy recommendation in 2007 or earlier because 1 was missing data and n = 400 after 2007 because 6 were missing data.

## Discussion

This study was designed to examine trends in behavioral and pharmacologic treatments and access to care in children ages 2 to 7 years with disruptive behaviors. In this study, we investigated a birth cohort and evaluated the differences in prescribing practices for children of different sex, race/ethnicity, SES, and insurance type. In regard to our first aim, we found that a child’s race/ethnicity, SES, and insurance differences did not affect their clinician’s psychotherapy treatment recommendations. It is noteworthy that although this sample came from a population with limited diversity, there were no substantial inequities in care.

For our second aim, we also sought to examine whether the implementation of treatment guidelines for young children described by AACAP in 2007 was reflected in the community over time (2008-2019). Contrary to our hypothesis, there was no significant change seen after 2007 in the frequency of psychotherapy recommendations before considering pharmacotherapy since the publication of the AACAP guidelines. Overall psychotherapy recommendations increased after 2007, especially for children to whom the 2007 article applied (<6 years old), but rates were similar among strict psychotherapy modalities. This might have been due to increased access to music, occupational, and other therapies, but this is unclear based on retrospective chart review. As treatment recommendations for children younger than 6 years almost universally include therapy, we also aimed to examine differences in psychotherapy recommendations for children ages 2 to 5 and 6 to 7 years. After 2007, the frequency of psychotherapy increased for children with ADHD younger than 6 years old. As with all age groups, recommendations for strict psychotherapy did not change, whereas rates for other and unspecified modalities were trending higher. From our third aim, we found that psychiatrists were more likely than PCPs to recommend psychotherapy (67% vs 48.4%). This finding remained even after adjusting for clinical risk factors and sex. Additionally, among children who were recommended therapy, patients were more likely to start therapy if recommendations came from a psychiatrist compared with a PCP (77% vs 69%). This combined to a total of 72% of children who started therapy.

Literature devoted to prescribing practices and trends over time in young children, especially in the context of specific child factors such as SES or demographics, is limited. Our findings suggest that among children for whom medications have been prescribed, race, SES, or insurance coverage did not affect treatment recommendations such as psychotherapy referrals provided by clinicians in the community. This is in contrast to other studies that showed that children with government insurance tend to receive medications at a higher rate than children with private insurance.^23^ Regarding race, White children are more likely to receive treatment for ADHD than Black and Hispanic children.^24^ A large, nationally representative study of schoolchildren showed that Black and Hispanic children were not only less likely to receive treatment but also less likely to be diagnosed with ADHD. Factors that contributed to lack of diagnosis were lack of insurance, single parent status, and parent unable to communicate in English.^24^ In contrast to some of the citied studies, the number of Asian, Black, and Hispanic patients was small in our sample.^23^^,^^24^ For this study we used data from a single population from the REP, which differs from other populations or the rest of United States. Based on data from 1970 to 2000, age, sex, and ethnic characteristics of residents participating in REP are similar to the state of Minnesota and the Midwest. At the same time, people from the studied population are less diverse, more educated, and wealthier than the overall US population.^25^

Young children are often referred to as therapeutic orphans because there are a limited number of randomized controlled trials that would effectively help in the creation of treatment guidelines.^26^,^27^ There are ongoing national shortages of psychiatric specialists for young children, often leading PCPs to provide care for these patients during brief appointments in busy clinics. A national study showed that 2 in 3 psychotropic prescriptions were written by PCPs.^28^ This is in line with our study, in which 68% of the children had their first psychotropic medication prescribed by their PCP or pediatrician, which would make the results from our third aim even more impactful. Moreover, recommendations for psychotherapy seem to be much higher in our sample size than in a prior study^29^ investigating national trends. Epstein *et al.*^29^ showed in a random sample of 50 pediatric practices that 93% of children diagnosed with ADHD were receiving medication and only 13% were receiving psychotherapy. In our study sample, 92% of children had an ADHD diagnosis, and 72% were receiving therapy after getting appropriate recommendations from their clinicians, with psychiatrists recommending therapy 67% of the time and PCPs recommending therapy 48% of the time. Interestingly, we also found that the young children in our study who had experienced trauma (61% vs 52%) and children with a family history of mental illness in a first-degree relative (59% vs 46%) were more likely to have psychotherapy recommended compared with children without such experiences or family history. While this can be attributed to a simple correlation, rather than a causation, perhaps treating clinicians of these children picked up on this and favored therapy as part of their trauma-informed care, for example, instead of relying on pharmacotherapy alone. This can also act as an introductory opportunity to further explore the root of the disruptive, externalizing behaviors of these children.

According to a study of older aged preschoolers by Garfield *et al.*,^30^ White boys had the highest likelihood of receiving psychotropic medication. Our study was not designed to find this association, as the inclusion criteria included that participants needed to be prescribed at least 1 psychotropic medication. Our data showed that psychotherapy was more likely to be recommended to boys than girls (56% vs 50%). More studies are needed to determine why boys were more likely to be referred to therapy. One theory could be that boys tend to demonstrate externalizing behaviors such as hyperactivity, impulsivity, aggression, and rule violation, whereas girls may have more internalizing problems.^31^ Similar to our study, medications for ADHD were the most common treatment. Almost 92% of our sample had a diagnosis of ADHD, and most were on a psychostimulant (90.5%). The prescribing practice trends did not seem to change despite the 2007 AACAP guidelines. PCPs are often left to prescribe medications due to lack of adequate access to psychotherapy resources and adequate time for collaboration with parents in treatment planning.^32^, ^33^, ^34^ Another study showed that after the American Academy of Pediatrics (AAP) guidelines in 2011 and AACAP guidelines in 2007, the rate of ADHD diagnoses in preschool-age children has slowed, but the number of children receiving medication has remained unchanged.^35^ Given significant shortages in mental health providers for children, most of the treatment for young children occurs in primary care clinics.^36^ More emphasis needs to be placed on providing appropriate education and resources in local community clinics. There may need to be more innovative approaches to care of young children with disruptive behaviors, such as collaborative care or integrative behavioral health models.

The present findings should be interpreted in the context of their limitations. Because this was a retrospective study, there was some level of variability in medical record documentation among different providers. We took care in classifying data. We had detailed chart information that allowed us to comprehensively assess the appropriateness of the diagnosis or treatment for the studied children. Most studies on young children report participant ages in months. We were unable to provide such detailed age as this would require both the birth date and the precise first prescription date, the latter of which could not be obtained accurately for all patients. Less than 15% of children’s medical records indicated cost and time commitment as reasons to not start psychotherapy, but the vast majority did not indicate a particular reason. Although the sample was representative of the Olmsted County population, the number of Asian, Black, and Hispanic patients was relatively small. The HOUSES index is a valid assessment of SES; however, a continuous measure might have provided greater precision and statistical power.

Results of this study suggest that children’s SES, race/ethnicity, and insurance did not affect treatment recommendations of clinicians. Children who had their first psychotropic prescription written by a PCP were less likely to have psychotherapy recommended compared with children whose prescriptions were written by psychiatrists. Additionally, patients were less likely to start psychotherapy if their psychotropic prescription was written by a PCP or pediatrician. Additional efforts are needed on a systemic and structural level to address this difference. It was encouraging to note that a child’s SES, race/ethnicity, and insurance did not affect their clinician’s treatment recommendations. Future efforts should focus on providing psychotherapeutic training and interventions that are harmonized with the needs of primary care clinics.

## CRediT authorship contribution statement

**Chris Wang:** Writing – review & editing, Writing – original draft, Data curation, Conceptualization. **Maria Saliba:** Writing – review & editing, Writing – original draft, Methodology, Data curation, Conceptualization. **Kierstin S. Utter:** Writing – review & editing, Data curation. **Joshua Wy:** Supervision, Funding acquisition, Data curation. **Alex S. Roth:** Writing – review & editing, Data curation. **Juan F. Garzon Hincapie:** Writing – review & editing, Data curation. **Tatsumi Yanaba:** Writing – review & editing, Data curation. **Pedro Versuti Del Cioppo Vasques:** Writing – review & editing, Data curation, Writing – review & editing, Formal analysis. **Vanessa K. Pazdernik:** Writing – review & editing, Methodology. **Chung-Il Wi:** Writing – review & editing, Supervision, Methodology, Funding acquisition, Conceptualization. **Monica J. Taylor-Desir:** Writing – review & editing, Writing – original draft, Supervision, Funding acquisition, Conceptualization. **Paul E. Croarkin:** Writing – review & editing, Writing – original draft, Supervision, Methodology, Investigation, Funding acquisition, Conceptualization.

## Figures and Tables

**Supplemental Figure S1:**