# Supplementary material for: Impact of Social Determinants of Health and Professional Guidelines for Pharmacotherapy and Psychotherapy Recommendations for the Treatment of Young Children: A Retrospective Study
Source: JAACAP Open. 2024 Dec 11;3(4):852–62. doi: 10.1016/j.jaacop.2024.09.010 (PMC12684468; doi:10.1016/j.jaacop.2024.09.010)
Supplement: Supplemental Table S2 [file mmc2.docx]

Supplemental Table 2: Multivariable logistic regression on psychotherapy recommendation based on prescribing physician’s medical specialty, childhood trauma, family history of mental illness, and sex (N=1712).

|  | **Psychotherapy recommended** | | **Strict psychotherapy** | | **Other therapy modalities** | | **Unspecified modality** | |
| --- | --- | --- | --- | --- | --- | --- | --- | --- |
| **Predictor** | **OR (95% CI)** | **P** | **OR (95% CI)** | **P** | **OR (95% CI)** | **P** | **OR (95% CI)** | **P** |
| Psychiatry | 2.03 (1.64, 2.53) | **<0.001** | 1.65 (1.33, 2.03) | **<0.001** | 1.61 (1.12, 2.30) | **0.01** | 1.10 (0.78, 1.55) | 0.576 |
| Male | 1.32 (1.06, 1.63) | **0.013** | 1.33 (1.07, 1.67) | **0.012** | 1.29 (0.87, 1.96) | 0.225 | 0.82 (0.58, 1.17) | 0.269 |
| Trauma | 1.23 (0.97, 1.55) | 0.083 | 0.94 (0.74, 1.19) | 0.616 | 1.03 (0.68, 1.54) | 0.878 | 1.84 (1.29, 2.61) | **<0.001** |
| Family history of mental illness | 1.55 (1.26, 1.91) | **<0.001** | 1.56 (1.25, 1.95) | **<0.001** | 1.14 (0.78, 1.70) | 0.51 | 0.96 (0.67, 1.38) | 0.81 |

Reference categories are Family medicine/Peds, Female, No trauma, and no family history of mental illness.

Strict psychotherapy included therapy modalities that were examined in paper by Gleason MM (2007) (behavioral management, PMT, PCIT, and CBT).

Other therapy modalities include those that were reviewed for their impact on early childhood psychiatric disorders (play therapy, parenting class, applied behavior analysis, social skills, occupation therapy, speech therapy, and music therapy).
